# Editorial: Breaks that benefit: international evidence to support innovation in research, policy, and practice around unpaid carer short breaks

**DOI:** 10.3389/frhs.2026.1932038

**Published:** 2026-08-18

**Authors:** Diane Seddon, Gillian Toms, Emma Miller, Elizabeth Hanson, Don Williamson, Kate Hogarth, Kim Whitmore

**Affiliations:** 1 School of Health Sciences, Bangor University, Bangor, United Kingdom; 2 School of Health Sciences, Bangor University Bangor Institute for Health and Medical Research, Bangor, United Kingdom; 3 Faculty of Humanities and Social Sciences, University of Strathclyde, Glasgow, United Kingdom; 4 Department of Health and Caring Sciences, Linneuniversitetet, Kalmar, Sweden; 5 Shared Care Scotland, Dunfermline, United Kingdom; 6 College of Nursing, Marquette University, Milwaukee, WI, United States

**Keywords:** caring relationships, respite care, short breaks, unpaid carer wellbeing, unpaid carers

Worldwide, increasing numbers of unpaid carers provide critical support to family members and/or friends who cannot manage independently due to illness, disability, mental health issues, frailty, or addiction. This increase is driven by the rising prevalence of chronic and degenerative conditions and population ageing, alongside the policy emphasis on care at home in the economically developed world and continued cuts to public services (1). International evidence confirms that caring for someone can be a rewarding experience (2), however, caring can also have significant negative impacts for unpaid carers' health and wellbeing (3, 4) that can lead to health and wellbeing inequalities.

Interventions to mitigate these inequalities are essential. Short breaks can play a key role in supporting carer wellbeing, reducing health and wellbeing inequalities and sustaining caring relationships. Unpaid carers identify access to short breaks taken *together* or *apart* from the person(s) they support as a priority and the form of support likely to make *the* most difference to their wellbeing when they are personalised to their individual needs, preferences, and desired wellbeing outcomes, and when carers have choice and control over their break options.

Many terms are used to describe breaks from caring. The most common terms are respite care and short break, and although they are sometimes used interchangeably, distinctions can be made. Respite is more aligned in some countries with traditional service-based breaks, which can be viewed as inconsistent with person centred practice. Increasingly, the term short break is associated with the advent of bespoke, more person-centred break options, and it refers to supporting carers and sustaining caring relationships through any type of service, assistance or activity that helps carers have time away from their caring responsibilities (5). These breaks often align with carers' personal interests and hobbies and reflect what matters to them.

Short breaks are a preventive intervention, and an important component of carer support strategies globally, aligning with the international policy commitment to support carers to live well. For example, a universal priority of the International Alliance of Carer Organisations recognises carers' right to “time off” from caring to maintain their own health (6). Article 24 of the Universal Declaration of Human Rights, United Nations (7), stipulates that everyone has the right to rest, leisure, and paid vacations, promoting fair working conditions and a balanced life.

This special edition shares new findings from international research conducted in the UK and America that contributes to our knowledge of short break needs, the preferences of unpaid carers regarding short breaks and short break outcomes. It enhances our understanding of what makes for a successful person-centred short break and how these breaks might support unpaid carers to realise important wellbeing outcomes in the short, medium, and longer term.

Two papers introduce a broader conceptualisation of short breaks as something that can be accessed outside of a “formal service”, and which could enhance person-centred practice. Utz et al. reimagine “respite” as something carers can create on their own in addition to its provision through formal break services. They found that during the COVID-19 pandemic when formal break services were not available carers used their daily activities and routines to take 'short breaks' throughout the day. As proactive citizens, they also drew on personal networks and shared caregiving arrangements to take informal breaks. Similarly, Weiss et al. found that following coaching, rural carers were able to access short tech-based breaks within their own homes to achieve greater respite and reduce their sense of burden.

Two papers explore how break benefits can be maximised. These papers speak to enhancing the co-planning by unpaid carers and practitioners that informs decision making at the centre of person-centred practice. Utz et al. found that setting goals for breaks and goal-review activities improved carers' subjective evaluation of their break time use. Further, Caulfield and Seddons scoping review findings underline the importance of regular discussions with carers about their break needs as they progress on their caring journeys. Early in the caring trajectory breaks that foster relational wellbeing may be especially valuable. In the later stages of caring, breaks that offer rest and recuperation may be called for.

In the fifth paper, Summers focuses on how to evaluate the outcomes of break services, highlighting the strengths and limitations of some of the available frameworks and models.

We hope this special issue will encourage knowledge exchange between academia, policy, and practice, help to shape and advance future research and encourage innovation in short breaks policy and practice development that will foster person-centred practice. You can get involved in this emerging agenda through the UK Short Breaks Research and Practice Development Group (email, d.seddon@bangor.ac.uk) and the international BREAK Exchange (email, kimberly.whitmore@marquette.edu).
